# Not All Forms of Severe Malaria Impair Long-term Cognition—More Characterization, More Questions

**DOI:** 10.1093/cid/ciaf713

**Published:** 2026-02-03

**Authors:** Karl B Seydel, Stephen T J Ray

**Affiliations:** Blantyre Malaria Project, Kamuzu University of Health Sciences, Blantyre, Malawi; Malawi-Liverpool-Wellcome Trust Clinical Research Programme, Blantyre, Malawi; College of Osteopathic Medicine, Michigan State University, East Lansing, Michigan, USA; Blantyre Malaria Project, Kamuzu University of Health Sciences, Blantyre, Malawi; Malawi-Liverpool-Wellcome Trust Clinical Research Programme, Blantyre, Malawi; Oxford Vaccine Group, Department of Pediatrics, University of Oxford, Oxford, United Kingdom; Institute of Infection, Veterinary and Ecological Sciences, University of Liverpool, Liverpool, United Kingdom

**Keywords:** severe malarial anaemia, cerebral malaria, severe malaria phenotypes, multiple seizures, neurocogntive impairment

**(See the Major Article by Bangirana et al on pages 1035–41.)**

Malaria causes an estimated 263 million cases and 597 000 deaths annually, with the World Health Organization’s (WHO’s) African region accounting for 94% of cases and 95% of deaths [1]. Children younger than 5 years experience the highest rates of severe malaria, and therefore the highest case fatality rates, and represent three-quarters of all malaria deaths. The WHO Intersectoral Global Action Plan on Epilepsy and Neurological Disorders [2] highlights the substantial neurocognitive burden among survivors of neuroinfectious diseases and call for greater evidence and rehabilitation capacity in high-burden, hard-to-reach low- and middle-income settings.

In the current issue of *Clinical Infectious Diseases*, Bangirana and colleagues [3] champion these WHO aspirations by building upon their group's prior studies evaluating neurocognitive outcomes in severe malaria. A heterogenous umbrella term, “severe malaria,” encompasses a collection of several distinct disease presentations with often overlapping and incompletely understood pathological processes. These include cerebral malaria (CM), severe malarial anemia (SMA), and malaria with additional severe criteria; respiratory distress (RD); multiple seizures (MS); and prostration (PR) [1]. Bangirana and colleagues have previously extensively reported high rates of long-term neurocognitive deficit in specific forms of severe malaria—both in its deadliest form, CM (mortality, 17%) [4], and its most prevalent form, SMA (1–5 million cases per year) [1]. In the present neurocognitive study, they extended their investigation to carefully characterize additional forms of severe malaria with RD, MS, PR, alongside CM and SMA.

The current study re-confirms their [5, 6] and others’ [7] prior prospective studies that CM and SMA are associated with impaired long-term neurocognitive outcomes. The novel findings are prospective evidence that the other forms of severe malaria—MS, RD, and PR—did not have any effect on long-term cognitive outcomes.

This well-designed study is a large prospective cohort (n = 600), in multiple sites, with rigorous a priori case definitions, well-matched contemporaneous controls, and robust statistical analysis, including Bonferroni correction. These multiple strengths enhance the reader's confidence when interpreting these novel findings, which are of public health importance considering the global burden of severe malaria.

The present study identified that neither SMA or CM was associated with attention or memory deficits. Curiously, while this reproduces prior study findings by this group (in this journal) [5] for SMA, both attention and memory deficits were identified after CM in the prior study. Several biologically plausible reasons for these important study outcome differences, such as age, sex, number of study sites, and number of malaria episodes during follow-up, were adjusted for by the authors (and remained nonsignificant in unadjusted analyses). Other potentially important differences, such as type of antimalarial therapy, could not be adjusted for in this study (artesunate was ubiquitous), but were not associated with attention and memory deficits in CM in their prior study (which overlapped the end of the quinine era). Furthermore, research methodologies between studies, such as neurocognitive testing instruments and study team supervision, were consistent between their studies. This leaves the reason or reasons for the CM behavioral outcome differences between studies unclear to both authors and readers.

Bangirana and colleagues logically structured the spectrum of severity of malaria phenotype (CM > RD > MS > SMA > PR) a priori in the present study, as defined by previous case fatality estimates. Yet, the current study findings offer a contrasting spectrum of neurocognitive severity (CM > SMA > RD/MS/PR). One might have previously assumed that PR, MS, and CM, each with neurological manifestations, are part of a neuropathophysiological and/or temporal disease continuum and, therefore, these 3 neurological phenotypes logically would confer the greatest brain injury in severe malaria, manifest with long-term neurocognitive insult. Yet, only SMA, with no characteristic neurological manifestations, alongside CM (the most severe neurological phenotype) were associated with neurocognitive deficit. These important and somewhat conceptually surprising findings shine a light once again upon our incomplete understanding of the neuropathobiology of brain injury in severe malaria. These important new neurocognitive findings across different severe malaria phenotypes should stimulate several next steps in this area. First, we encourage further longer duration neurocognitive and behavioral assessments and characterization in such populations, not least as prior studies in CM illustrate very late onset neurological impact post-CM [8]. This may also disentangle the current equipoise of behavioral outcomes post-CM.

A recent study [9] showed that SMA and CM have distinct gut microbiomes yet share some gut bacterial pathogenic dysbiosis related to death. This offers further “organ specific” insight into diverse severe malaria biology, to echo the current study’s heterogenous neurocognitive findings across different severe disease phenotypes. Most crucially, these study data raise new key neurobiological questions and should be a catalyst into investigating the mechanistic pathways of brain injury across different forms of severe malaria. These methods could be multimodal, including blood–brain barrier and neuro-microvascular models, high-quality contrast imaging, and serial soluble neuronal injury markers, to guide improved interventions to reduce hypoxic and cytotoxic injury and consequent neurocognitive deficits. Collectively, the present study illustrates the value of rigorous, systematic characterization of severe malaria, illuminating that both clinical phenotypes and outcomes are not uniform. Fundamentally, the careful study characterization infers that *Plasmodium falciparum* likely drives diverse neuropathogenic pathways that underpin the disparate clinical presentations and neurological burden of severe malaria, but more mechanistic understanding is urgently needed.
